# A quantization assisted U-Net study with ICA and deep features fusion for breast cancer identification using ultrasonic data

**DOI:** 10.7717/peerj-cs.805

**Published:** 2021-12-16

**Authors:** Talha Meraj, Wael Alosaimi, Bader Alouffi, Hafiz Tayyab Rauf, Swarn Avinash Kumar, Robertas Damaševičius, Hashem Alyami

**Affiliations:** 1 Department of Computer Science, COMSATS University Islamabad-Wah Campus, Wah Cantt, Pakistan; 2 Department of Information Technology, College of Computers and Information Technology, Taif University, Taif, Saudi Arabia; 3 Department of Computer Science, College of Computers and Information Technology, Taif University, Taif, Saudi Arabia; 4 Department of Computer Science, Faculty of Engineering & Informatics, University of Bradford, Bradford, United Kingdom; 5 Department of Information Technology, Indian Institute of Information Technology, Uttar Pradesh, Jhalwa, Prayagraj, India; 6 Faculty of Applied Mathematics, Silesian University of Technology, Gliwice, Poland

**Keywords:** Quantization, Features fusion, Breast cancer, Ultrasonic images, Computer vision, Image processing

## Abstract

Breast cancer is one of the leading causes of death in women worldwide—the rapid increase in breast cancer has brought about more accessible diagnosis resources. The ultrasonic breast cancer modality for diagnosis is relatively cost-effective and valuable. Lesion isolation in ultrasonic images is a challenging task due to its robustness and intensity similarity. Accurate detection of breast lesions using ultrasonic breast cancer images can reduce death rates. In this research, a quantization-assisted U-Net approach for segmentation of breast lesions is proposed. It contains two step for segmentation: (1) U-Net and (2) quantization. The quantization assists to U-Net-based segmentation in order to isolate exact lesion areas from sonography images. The Independent Component Analysis (ICA) method then uses the isolated lesions to extract features and are then fused with deep automatic features. Public ultrasonic-modality-based datasets such as the Breast Ultrasound Images Dataset (BUSI) and the Open Access Database of Raw Ultrasonic Signals (OASBUD) are used for evaluation comparison. The OASBUD data extracted the same features. However, classification was done after feature regularization using the lasso method. The obtained results allow us to propose a computer-aided design (CAD) system for breast cancer identification using ultrasonic modalities.

## Introduction

Many cancer diseases have been found in the last couple of decades, and breast cancer is the most prominent. It is produced in the breast region and spreads through the tissue. The normal growth of tissues is not dangerous for the human body; however, due to medical reasons, abnormal tissue growth occurs. It harms the body by spreading over other organs (Latif et al., 2020). New cases of breast cancer reported worldwide in 2020 are 2.26 million. The woman ratio of total cases is nearly 99%. In 2020, 0.68 million deaths due to breast cancer were reported globally (Sung et al., 2021). However, the risk can be reduced using advanced strategies to diagnose it at its early stage accurately (Hussain et al., 2020; Latif et al., 2020; Li et al., 2020; Pang et al., 2020; Pi et al., 2020).

The lesion in the breast region is challenging to find due to its complex intensity ranges. Additionally, most females experience failure in their diagnosis. The lesions in the breast region have similarities with other breast organs due to their intensity, texture, and other morphological patterns (Hussain et al., 2020). Therefore, lesion segmentation from the breast region has become a challenging task. The benign and malignant tumors contain different morphological features. Many recent studies Smistad et al. (2015), Wang, Cui & Zhu (2020b), Huang, Luo & Zhang (2017), and Shen, Wu & Suk (2017) have been proposed for breast tumors identification where benign tumors, in most cases, are round or oval, have less density, and have a certain margin value and the malignant tumor is an abnormal tumor with a non-regular shape and highly dense characteristics (Li et al., 2020). Many imaging modalities are used for the diagnosis of cancers, and common ones include Magnetic Resonance Imaging (MRI), mammography (X-rays), and ultrasonic imaging (sonography) (Latif et al., 2020). They also reduce false positives and the false cancer detection ratio (Catalano et al., 2019; Carlino et al., 2019; Alikhassi, Azizi & Ensani, 2020). Mammography is considered the most effective modality for breast cancer diagnosis, but it lacks effectiveness for dense breast masses and low sensitivity for females under 40 years. The sensitivity for breast masses can be covered using an ultrasonic modality. The ultrasonic modality has other benefits: a lower cost, a quick acquisition, more subjectivity, and non-invasiveness (Pi et al., 2020). The ultrasonic modality uses sound waves that are not harmful to body organs, whereas X-rays use high-risk waves. For breast cancer diagnosis, the sound wave frequency range is set to 30–60 MHz, and this range is normally between 2 and 15 MHz for other tests (Latif et al., 2020).

Several public datasets for ultrasonic breast modalities are available for diagnosis; the most prominent are OASBUD Piotrzkowska-Wróblewska et al. (2017), and BUSI Al-Dhabyani et al. (2020). To utilize the effectiveness of fair clinical information, the proposed method used an ultrasonic imaging dataset. Experts of ultrasonic imaging usually evaluate reports using their geometry, density, and internal echo contrast levels (Sultana et al., 2020). The manual evaluation can be wrong due to a non-experienced sonographer or many other real-time problems. Therefore, an automated and accurate system needs to exist to help sonographers to more confidently perform cancer diagnosis (Pi et al., 2020; Sultana et al., 2020).

The proposed approach contains two significant phases of segmentation and classification. U-Net is a contraction–expansion approach that is considered to be the most effective semantic network for segmentation (Vakanski, Xian & Freer, 2020). In this research, a two-stage segmentation of breast tumors is utilized:

1. The precise, two-step isolation of the breast lesion is performed with Quantization assisted U-Net on sonographic images;
2. ICA-focused features are extracted and fused with Densenet201 features to achieve a highly promising identification of breast cancer.

This article is further divided into four parts: Related Work, Proposed Method, Results and Discussion, and Conclusion.

## Related work

Deep learning is one of the most commonly used techniques in the modern era to solve recognition-related medical tasks, among other tasks in the field (Albahli et al., 2021b; Albahli et al., 2021a; Meraj et al., 2020). There are many other smart city automation (Gheisari et al., 2021), resources allocation optimization Malik et al. (2021), renewable energy stability (Gao, Wang & Shen, 2020b) where ML (Gao, Wang & Shen, 2020a) and DL (Gao, Wang & Shen, 2020c) based task failure prevention for cloud based data-centers provide an intensive look to adopt intelligent solutions in each aspect of life. Similarly, time forecasting based data is also used to predict alarming situations (if any) such as COVID-19 creating a global emergency in the world that needs to be predicted before time in order to take corresponding initiatives (Rauf et al., 2021; Namasudra, Dhamodharavadhani & Rathipriya, 2021). Many studies use U-Net for semantic information, which later yields spatial information for contextual level set methods to obtain feature maps. There are more discriminative results for ultrasonic tumor segmentation in three datasets (Hussain et al., 2020).

A Gauss-Newton inverse algorithm was used for the reconstruction of both ultrasonic and dielectric datasets. This quantification has been reported as a helpful approach for the identification of tissue-type breast lesions (Mojabi, Khoshdel & Lovetri, 2020). Other studies have used lymph nodes by proposing a nomogram that depends on breast lesion features. Different features, including geometric features, such as shape, orientation, and boundary, and other features, such as vascularity and pattern echo, are used.

The best AUC value was achieved using boundary and size features (Luo et al., 2020). Another study used a hybridized transfer learning-based method for breast cancer recognition from the same trained deep convolutional neural network (D-CNN). The patch-wise classification was reported as less accurate than class-wise classification accuracy, yielding 90.5% and 97.4%, respectively. Patch-wise classification was performed using four classes: benign, normal, *in-situ*, and invasive-carcinoma, and class-wise classification was performed as benign or malignant (Alzubaidi et al., 2020).

Radio-signal-based ultrasonic breast imaging data is also available for the classification of breast cancer. Another study utilized the amplitude value of radio signals with the help of a D-CNN. This study reported a good classification performance with an AUC of 0.77 and an accuracy of 0.710. Moreover, it used the nakagami parameter in classification, which achieved a 0.64 AUC and an accuracy of 0.611 (Jarosik et al., 2020).

Another study used a super-pixel approach with ROI cropping and then performed preprocessing operations such as a bilateral filter, histogram equalization, and shift filtering of the mean type. The simple linear iterative clustering-based super-pixel method was then performed on preprocessed, enhanced patches. Next, a GLCM (grey level co-occurrence matrix) was used with a grey histogram and local binary patterns (LBPs) for feature extraction. At last, classification and re-classification were performed using K-means and KNN algorithms, respectively (Huang et al., 2020).

Deep features have played an important role in the progress of deep learning. These in-depth features are extracted from the deep convolutional layers of pre-trained CNNs or custom-designed CNNs. One study used transfer learning on a pre-trained CNN and trained it using BUS data containing 2,099 images. This dataset was collected from 543 patients with tumors, 302 of which were benign, and 241 were malignant. An SVM with a sequential optimization solver was used for classification (Shia & Chen, 2021). The BUS images contained speckle noise. A preprocessing phase of fuzzy enhancement, blur edge, and bilateral filtering was performed to improve this noise. Three model features have been used in transfer learning, where adaptive spatial feature fusion was performed for the feature selection approach. A dataset of 1,328 images was used (Zhuang et al., 2021). Another study stated that the lesion area in BUS images needs more attention to obtain useful information from the lesion’s marginal zone and posterior echo region. The in-depth feature fusion of all these different types of ROI information needs to fuse with the original image to obtain a more confident diagnosis (Yu et al., 2021).

A radionic approach using many features has been proposed, where the shape, a histogram gradient, and texture features were considered the most significant values to diagnose breast ultrasound images. It achieved an accuracy of 97.4% (Mishra et al., 2021). Similar to this radiomic feature approach, another study used content-based radiomic features from the BUS images. This study considered 895 breast ultrasound images and used a color histogram; texture features using the Haralick method, and Hu Moment features. It also proposed a CNN architecture to classify benign and malignant types Wan et al. (2021). An attention module was integrated into the VGG16 model, which improved the original VGG16 model performance and showed an accuracy of 93%. It also suggested that the loss function should be topic-specific (Yousef Kalaf et al., 2021). Another deep learning-based approach using DenseNet121 was proposed as an attention model, which used 785 BUS images collected from 367 patients. Two models with fine and coarse ROIs were proposed. They achieved AUC values of 0.87 and 0.90, respectively (Dong et al., 2021). A generative model to classify breast and thyroid cancers has been proposed. Although it was different from that of other studies, the framework proposed was considerable. It used 719 images of the thyroid, including 672 breast images. A deep CNN (DCNN) was proposed and showed an 86.5% accuracy for ThoridNet and an 85.9% accuracy for Breast-Net. It classified two types of cancer, each showing benign and malignant cases (Zhu et al., 2021). Some related work studies are summarized in Table 1 with their methods, dataset, and results in detail.

**Table 1:** Summary of recent studies on ultrasonic breast cancer identification.

Reference	Method	Year	Dataset	No. of images	Purpose	Results and evaluations
Huang et al. (2020)	Super-pixel based semantic segmentation	2020	Private	Total = 320		
				M = 160		
				B = 160	Segmentation	F1-score = 89.87% ± 4.05%, A- radial error = 9.95% ± 4.42%
Zhou et al. (2021)	3D image segmentation based upon CNN and classification upon probability	2020	ABUS	170 Volumes	Segmentation and Classification	Dice = 0.778, Jaccard = 0.65 and Acc = 0.74, recall = 0.882, prec = 0.83, F1-score = 0.811
Wang et al. (2020a)	Multi-view CNN with transfer learning	2020	ABUS	Total = 316		
				M = 135		
				B = 181	Classification	Sensitivity = 0.89, specificity = 0.87
Pang et al. (2021)	Augmented data with semi-supervised GAN network	2021	Private	Total = 1,447		
				M = 680		
				B = 767	Classification	Acc = 90.41, sensitivity = 87.94, specificity = 85.86
Shia & Chen (2021)	Pre-trained CNN with SVM	2020	Private	Total images = 2,099		
				Patients = 543		
				M = 241		
				B = 302	Classification	Sensitivity = 94.34, Specificity = 93.22, AUC = 0.938
Kriti, Virmani & Agarwal, 2020	Adaptive-neuro fuzzy classification with pre-trained-model-based feature extraction	2020	Sonoskills and hitachi med sys. Europe	Total = 100		
				M = 60		
				B = 40	Segmentation and Classification	Acc = 98, AUC = 0.983
Wei et al. (2020)	Texture and morphological feature-based classification	2020	Private	Total = 448		
				M = 264		
				B = 184	Classification	Acc = 91.11, sens = 94.34, spec = 86.49
Liang et al. (2020)	Texture Analysis on BUS images	2020	Private	Total = 85		
				M = 35		
				B = 50	Classification	Sens = 0.77, spec = 0.84, acc = 0.81, ppv = 0.77, npv = 0.84, kappa = 0.61

We can see in Table 1 that most of the recent studies using ABUS and privately created datasets by their local or national labs are composed of benign and malignant patient data. It also shows that some of them only focus on segmentation and classification, while most studies focus on classification and segmentation separately. However, the proposed method employs both segmentation and classification for precise and promising results.

## Methodology

**Figure 1:**
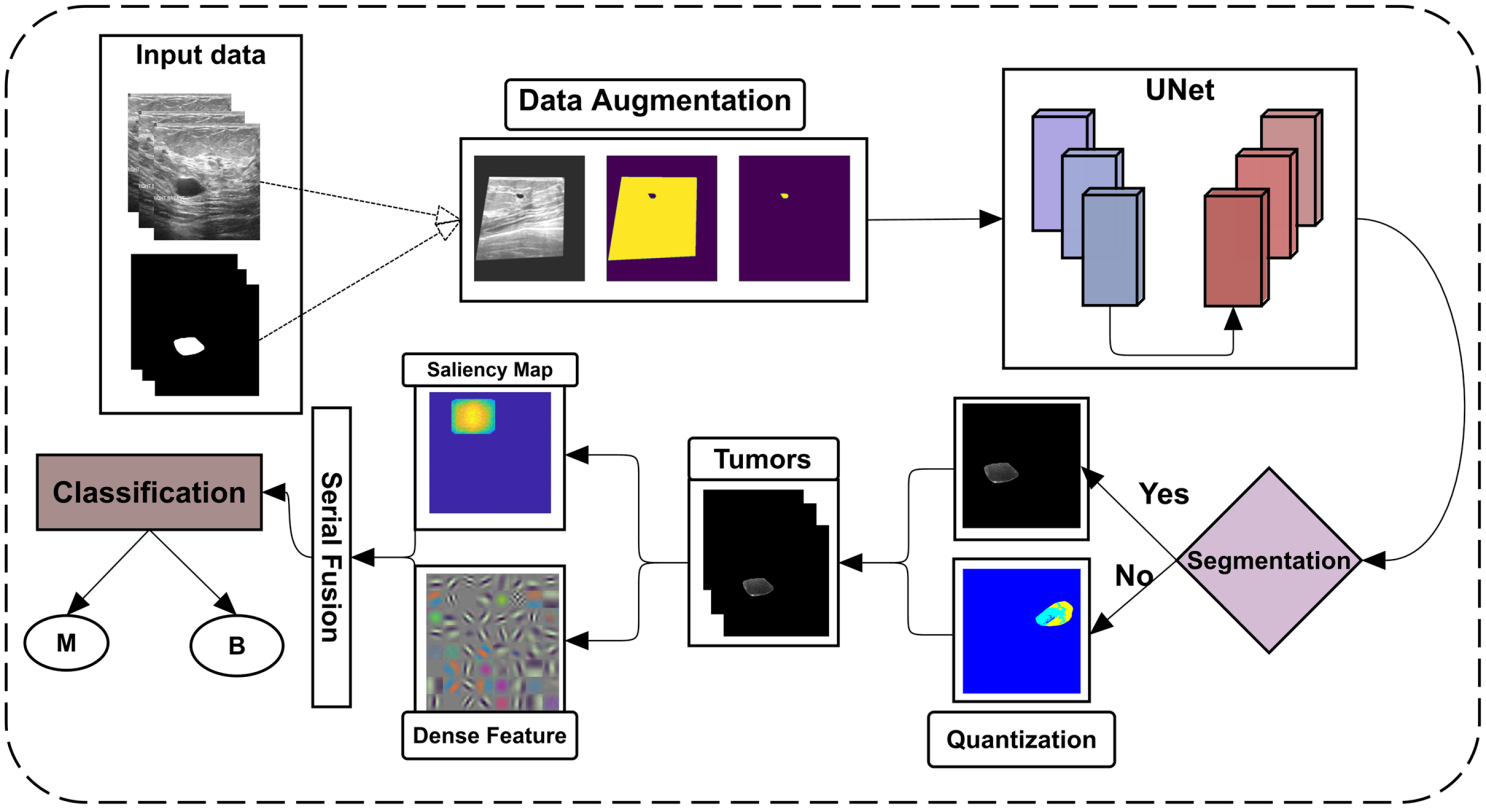
Flowchart of the proposed framework.

The proposed approach detects and identifies breast tumors from ultrasonic images; all primary steps are shown in Fig. 1.

The BUSI dataset images and their ground-truth labels are firstly passed from the data augmentation step, which U-Net further uses for training. The U-Net model is then tested on the testing dataset of BUSI and another dataset, OASBUD (Open Access Database of Raw Ultrasonic Signal Breast Lesions). After getting U-Net segmentation results, some lesions are not accurately segmented; quantization is then performed to reconsider them to extract lesions. By obtaining all lesions, the proposed method extracts the ICA features from the lesions, which are later fused with deep features extracted using DenseNet201. These fused features are then fed to different machine learning classifiers using 5- and 10-fold cross-validations to perform classification.

### Data augmentation

The ultrasonic data are used with their labels to process the data. In the dataset, images are not equally sized. Therefore, BUSI data are approximately 512 × 512 × 3 and are resized using the Nearest-Neighbor method. The resizing needs geometric operations to remap the given digital lattice. For example, if the given image is represented as I, then an operation is performed on the image using Eq. (1), where Eq. (2) is representing the *f*(*n*):

(1) $$I\left( n \right) = f\left( {{n}^{\prime}} \right) = f(a(n))$$

where *f*(*n*) is further representing *a*(*n*) operation, the *f*(*n*) working according to Eq. (2).

(2) $$f\left( n \right) = \left[ {INT\left( {{a_1}\left( {i,j} \right) + 0.5} \right)} \right],\left[ {INT\left( {{a_2}\left( {i,j} \right) + 0.5} \right)} \right]$$

**Figure 2:**
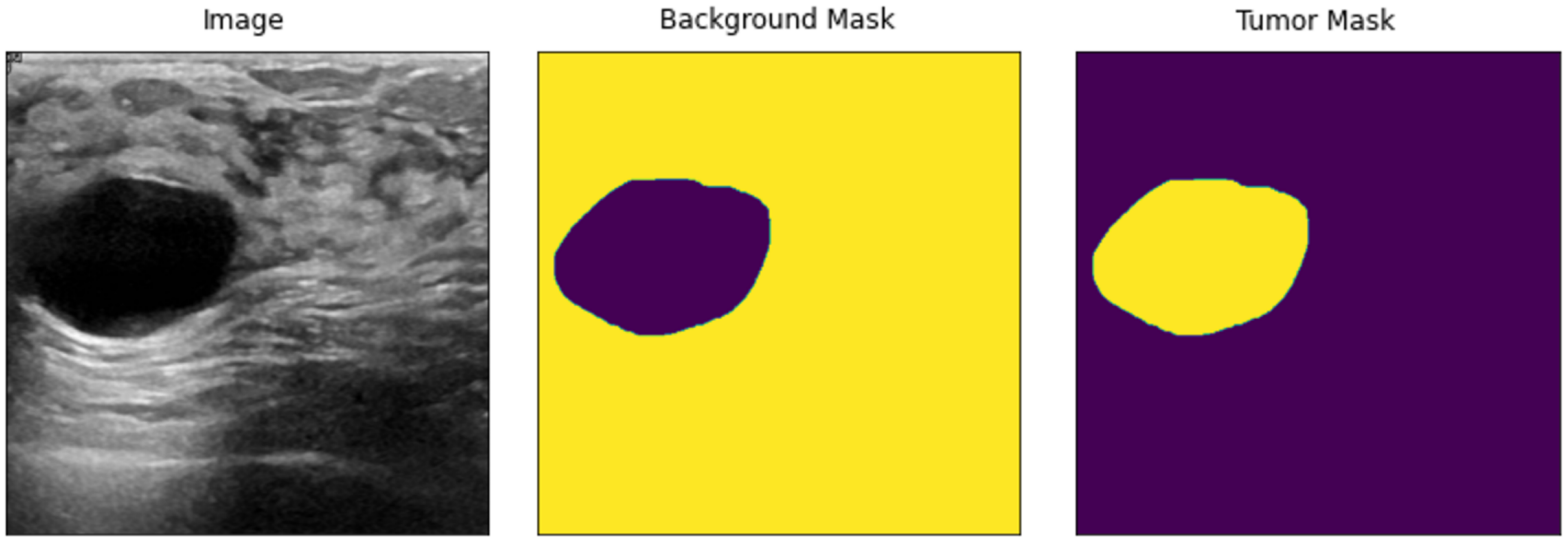
Resized input samples (left) = original Images, (Mid) = background mask and (right) = tumor mask.

In Eq. (2), the lattice is handled with two new values as a single value of *a*_1_, which does not entirely represent a newly defined image due to spatial operations, as the new lattice contains a decimal place value. Therefore, two values of *a*_1_ and *a*_2_ are combined to define the new size of the image. The input images are then resized using the explained method or operations. The new input images are shown in Fig. 2.

**Figure 3:**
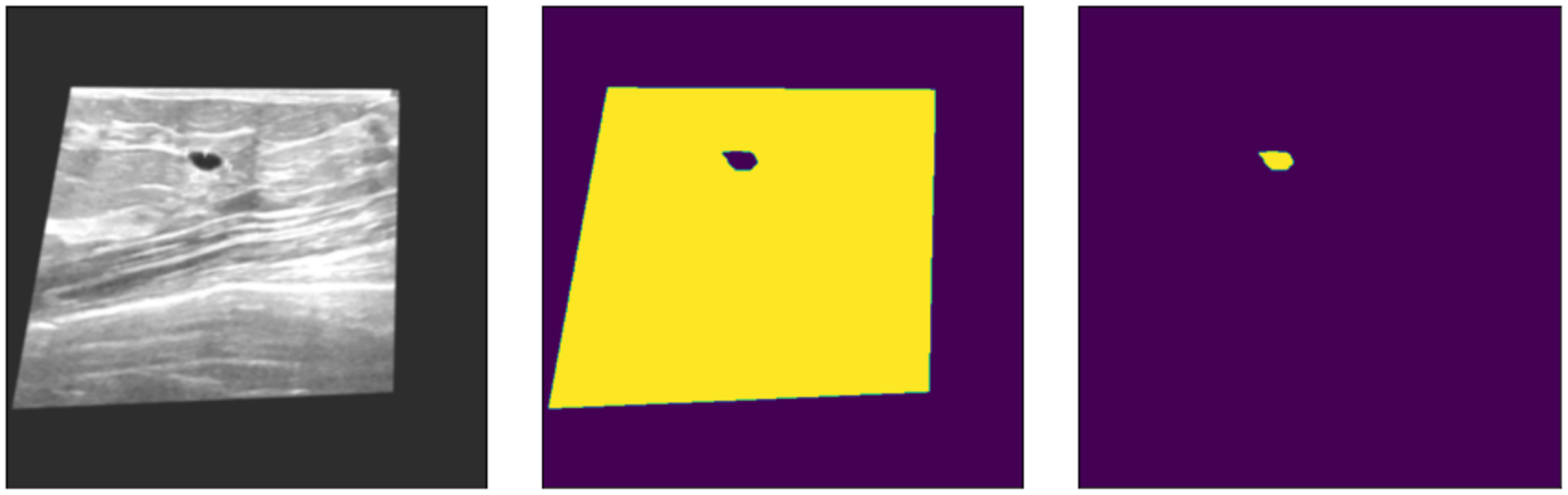
Augmented data samples. Left = pre-processed input image; Mid = pre-processed tumor mask; Right = pre-processed input image background.

After resizing, all the given ground truth labels and input images become equal, as shown in Fig. 2, but preprocessing is still needed to feed the input data to U-Net. To remove speckle noise, the input image regions need to be enhanced. Data augmentations are used to handle the overfitting problem in the deep learning models, where more input instances of data samples are created. Therefore, the proposed method uses fast and very flexible image augmentations on the dataset. According to Buslaev et al. (2020), the images are transformed w.r.t. given dimensions using copping and padding, if needed. Almost all of the data preprocessing methods are available; the user chooses operations according to the acquired data. The resized input data are clipped with a shift scale rotation with horizontal flipping. Similarly, we also added contrast stretching, brightness adjustments, sharpening, and contrast-limited adaptive histogram equalization (CLAHE) to used datasets. The overall data are transformed, enhanced, and ready to solve the overfitting problem of deep learning models. The augmented data have a much better resolution and clearance, as shown in Fig. 3.

As shown in Fig. 3, the data and its corresponding labels are also shifted to the same angle and rotation so that images remain correspondent to each pixel label. Thus, the augmented data do not lose any spatial information of the original input images and masks. Furthermore, it also enhances the input samples and removes the speckle noise from the images, if any. However, these preprocessed augmented input data are fed to U-Net for the semantic segmentation of the BUS imagess.

### U-Net-based segmentation

**Figure 4:**
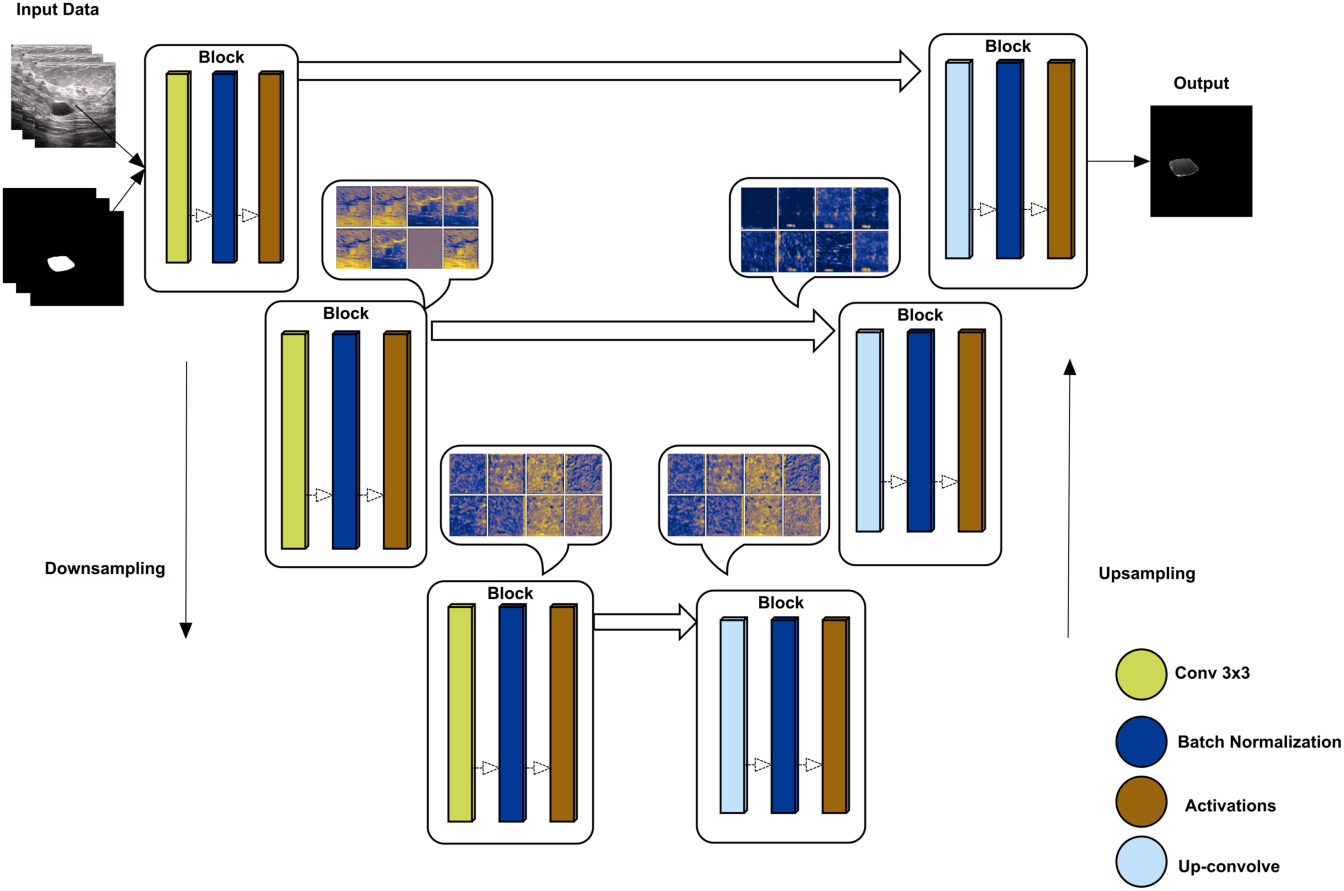
U-Net architecture using downsampling and upsampling approaches (backebone = inceptionrenset-v2) with a convolutional block activation sample of the proposed study data.

U-Net has an encoder-decoder approach to semantically segment the images (Yakubovskiy, 2019). The encoder side of the model down-samples to the given images with their labels, while the up-sampling approach re-codes the images to decode the image back to reach the final convolutional layer. The proposed method uses tensor-flow as a helper library to obtain a model of U-net where the backbone is ‘inceptionresnetv2’, which is used to train using the given augmented data. inceptionresnetv2 is selected for its high accuracy rate compared to other backbones. The overall architect of U-Net is shown in Fig. 4 with some convolutional block visualization. In the inceptionresnetv2 backbone, the model has multiple blocks, including three blocks of convolution, batch normalization, and activation, where pooling is performed using the max and average of various blocks.

**Figure 5:**
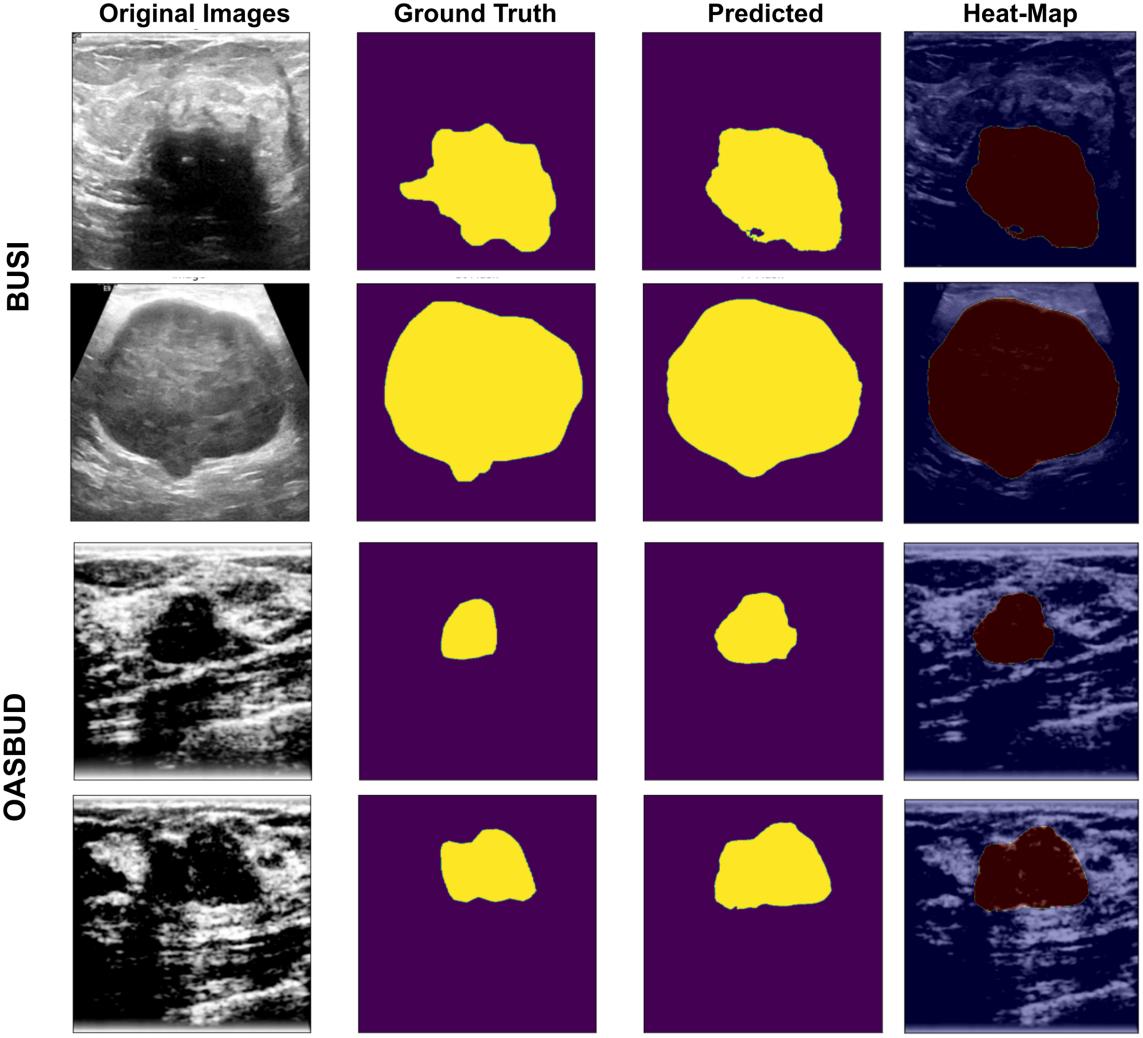
U-Net-based segmentation on the BUSI and OASBUD datasets. Column-1 = Original Images; Column-2 = ground truth masks; Column-3 = U-Net predicted masks; Column-4 = Activated heat maps of Predicted area on input images, where the first two rows represent the BUSI data samples, and the last two rows represent the OASBUD data samples.

The U-Net-based segmentation was tested on two datasets, and the results are shown in Fig. 5. The OASBUD dataset images are noisy, as raw image inputs are not the same as BUSI images. Therefore, using enhancement techniques, the dataset was first processed using a Gaussian smoothing filter and histogram equalization and then passed through a data-augmentation process explained in the first sub-section of the Methodology section.

(3) $$S(i,j) = \sum\limits_{i = 1}^n \sum\limits_{j = 1}^n {\rm ex}{{\rm p}^{ - \displaystyle{{{i^2} + {j^2}} \over {2{\sigma ^2}}}}}$$

(4) $${S_1} = \displaystyle{1 \over {2\pi {\sigma ^2}}}*S(i,j)$$

In Eqs. (3) and (4), Gaussian blur is explained where I and j are the rows and columns of any given digital lattice, where *σ*^2^ represents the standard deviation, which is taken as 0.5 in experimentation. This exponentially powered result is later on multiplied with a denominator of the product of 2*π* and *σ*^2^. It returns a final smoothed image in the case of OASBUD images that contain noise. Later on, histogram equalization is applied to the image, which can be calculated in Eqs. (5)–(7).

(5) $$H = T(h)$$

Eq. (5) explains the transformed histogram for a given image, which is our smoothed image in the experiment, so we can make Eq. (6), which shows the equally distributed gray-level intensity bins of the given smoothed input image.

(6) $${S_{{1_T}}} = T({S_1})$$

This transformation occurs with a cumulative histogram subtraction, as shown in Eq. (7).

(7) $$E = |{c_i}\left( {{S_{{1_T}}}} \right) - {c_0}({S_1})$$

Eq. (7) explains that the cumulative histogram of the transformed bins is subtracted from the input image grayscale bins, showing the final enhanced image ‘E’, used later for segmentation testing purposes.

As we can observe in Fig. 5, Column-1 contains input images of testing data of the BUSI and OASBUD datasets. The 2nd column contains the input ground-truth masks of both datasets. The 3rd column shows the U-Net predicted lesion masks of input images of Column-1. The last column shows the predicted lesion-based heat-map visualization of the input images. It localizes the lesion part of the input images for better interpretation. These lesions are well-segmented, but some of the images were not extracted as required. Those segmented lesions were re-considered for segmentation using the quantization process.

### Quantization based on Otsu’s method

**Figure 6:**
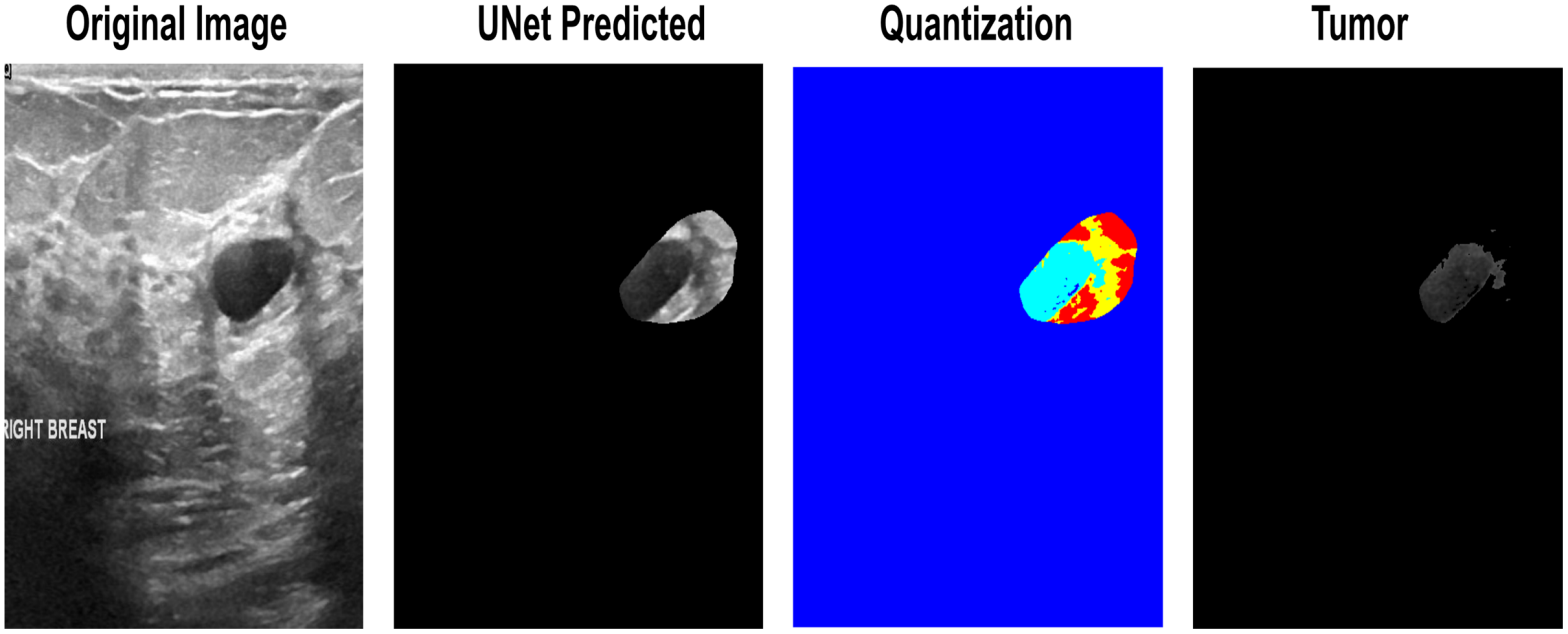
The quantization-based re-segmented tumor. 1. Input Image. 2. Imprecise Prediction of U-Net. 3. Quantization. 4. Isolation of the exact lesion area.

While testing datasets, some of the images were not entirely satisfactory in determining the exact lesion area. First, the lesions obtain a threshold using Otsu’s threshold method and return a 1 × *n* vector, which can be used to obtain *n* + 1 discrete values from an image further. These vector-based indexes make quantized images from which the tumor-level-based selection is performed to finalize the input lesion. One of the input images that needs reconsideration for segmentation is the quantization-based selection of the lesion area in Fig. 6.

The final quantized lesions and U-Net-based segmented lesions are then used to extract ICA and denseNet201 deep features to use separately and in serial fusion to classify the breast lesions.

### Independent component analysis features

**Figure 7:**
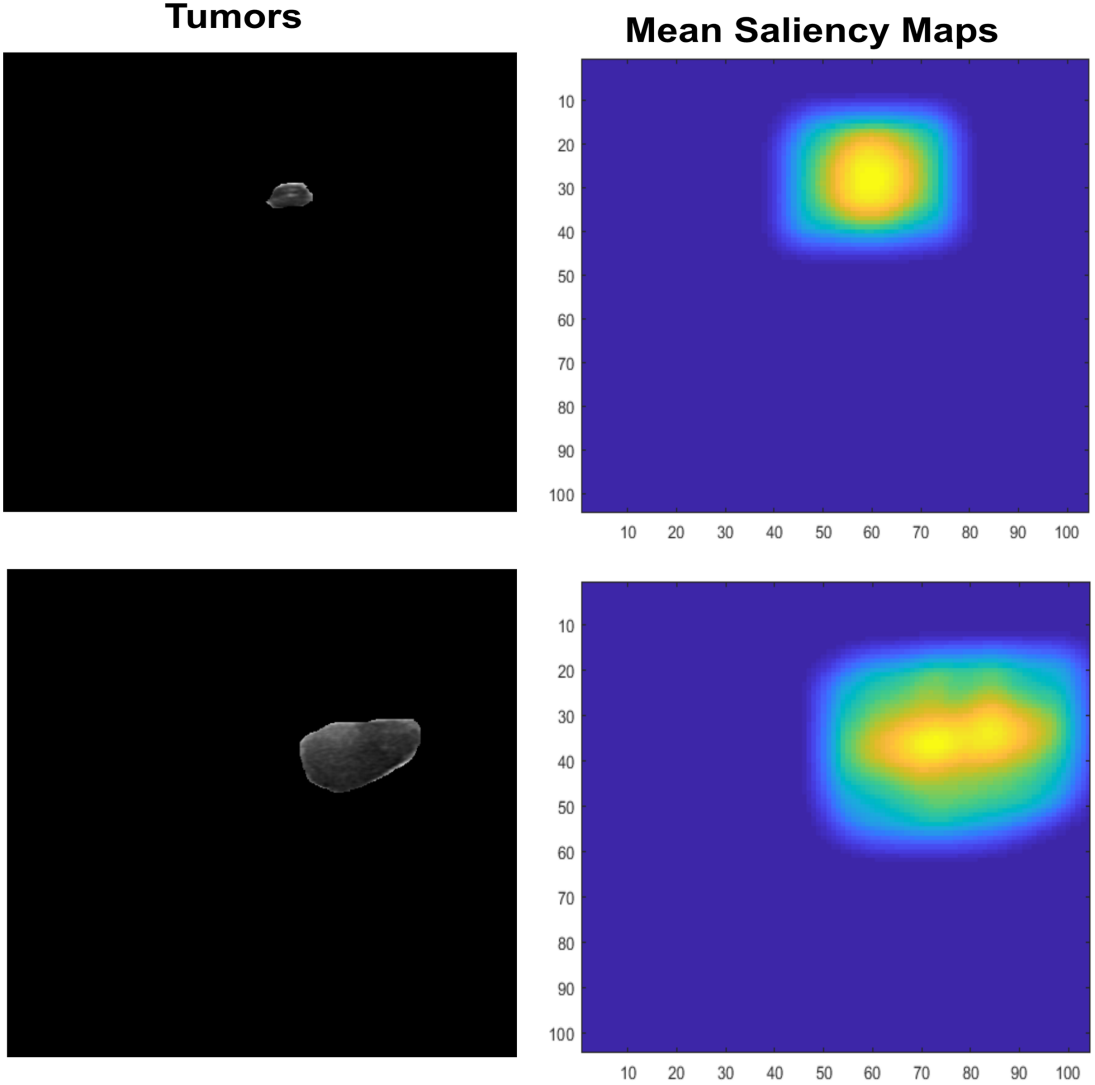
The input lesion images (left) and mean saliency maps (right).

Features Extraction is an important that leads to higher rates or prediciting accuracy, it plays main role in classification. Many recent studies use them in various aspect of field to recognize the given objects such as abstract art painting based features recognition (Li, Raj & Namasudra, 2021). However, Saliency maps are nature inspired, biological plausible features. It calculated as natural statistics in image. Many features are used for recognition or classification tasks but those are not so generalized for visual tasks, also image classification needs visual ability to cope with partial views. Therefore, inspiring from saliency visual statistics (Ramanishka et al., 2017) is used in proposed study. The Independent component analysis (ICA) features are firstly extracted. The sparse filters are created which contain luminance in it, also have effects of chromatic properties. At first, the small patches are extracted with LMS (long, medium, short) color space channel means subtraction. These patches are further used by PCA to reduce its dimensionality, also the high Eigen value containing responses are excluded. The finally normalized feature vector ‘*f*’ is used by Saliency using natural statistics (SUN) framework to make saliency maps which further taken as responses means and shown in Fig. 7.

The SUN framework uses the bottom-up saliency type to make a map. This saliency is defined as *P*(*f*)^−1^, where the features are represented by *f*. As the dimensions of the feature vector increase, the ICA becomes more statistically independent. Therefore, the dimension of one-directional distributions can be written as P (F). Both are represented in Eqs. (8) and (9).

(8) $${({f_{}})^{ - 1}}$$

(9) $$P(F) = {\pi _i}\;P({f_i})$$

In Eq. (9), the *f*_*i*_ representing the features vector is explained above, where *P*(*f*_*i*_) represents the generalized Gaussian distributions used as a one-dimensionality conversion method.

#### Deep dense features

Deep learning is used by much recent recognition and classification tasks that are extensively inspiring real-world problem solutions (Lal et al., 2021; Rehman et al., 2021). To utilize that deep learning approach, one of the pre-trained models named denseNet201 is used. Fully connected layer-based transfer learning is applied, and *f*_2_ is created. It is individually used for classification and for serial fusion.

#### Serial fusion

Features fusion using hybrid approaches such as deep features in various medical imaging diagnostic field is using now a days such as COVID-19, Knee Osteoarthritis (Mahum et al., 2021), Diabetic retinopathy (Lal et al., 2021), extracted using different features fusion approaches. However, ICA response-based normalized feature *f*_1_ and the deep transfer learning-based feature vector *f*_2_ are used in proposed study to fuse using serial method. The third feature vector named *α* contains the sparse statistical information of segmented lesions with deep, dense, automatic features. This *α* vector-based classification is also used for benign and malignant lesion classification.

(10) $$\alpha = (f_1 + f_2) - n \times m\ dimensional

The mathematical representation of the serial formation of used features in the proposed methodology is expressed. The ‘n × m’ representing the n-dimension of *f*_1_ and m-dimension of *f*_2_.

### Classification

The classification of malignant and benign cases of the used ultrasonic imaging is used with two validation techniques, 5-fold and 10-fold. Different 6-type classical machine learning methods are used and include two variants of support vector machine (SVM), named Cubic-SVM (C-SVM) and Quadratic-SVM (Q-SVM). Moreover, the ensemble bagged-boost (Bag-Boost), RUS-boost (R-Boost), and medium tree (M-Tree) are used. At first, the effect of the saliency-based feature is checked. At the 2nd experiment, the deep, dense features are serially fused to obtain the combined effect of both features to yield more confident results.

## Results and discussion

The proposed method mainly uses two types of approaches to feature selection for the sake of classification. The first section contains 5- and 10-fold results on mean saliency features with five classification methods, and the second section serially fuses dense features with 5- and 10-fold validation methods using the same five classification methods.

### Dataset description

Two publically available datasets were employed to evaluate the proposed approach. Dataset-1 uses the BUSI dataset, which contains three types of images—normal, benign, and malignant. The U-Net model is trained on the BUSI training images, which are tested on two datasets. The two testing datasets contain data from the BUSI testing set and OASBUD-enhanced images, respectively. The BUSI dataset was available in images format where OASBUD data was available in raw format. However, the given data is used to create images and their corresponding masks. The multiple morphological operations using its features such as area, circularity, roundness etc. These features are used to get contours of this data. However, the enhancement operation is also performed on OASBUD data using same methods as used in BUSI data, Both dataset descriptions are shown in Table 2.

**Table 2:** Datasets descriptions.

Classes	BUSI	OASBUD
Benign (B)	437	48
Malignant (M)	210	52
Total	647	100

### U-Net experimental setup

The first step of deep-learning-based segmentation is performed using U-Net on two classes, background and tumor. The number of epochs is increased and decreased, which affects the final results. Therefore, 40 epochs are taken as the final optimal number of epochs. However, the learning rate is set to 0.0001. All other training parameters are shown in Table 3.

**Table 3:** U-Net training parameters.

Parameter	Value
Backbone	Inceptionresentv2
Learning-Rate (LR)	0.0001
Epoch	40
Activation	Sigmoid
Batch-size	8
Training environment	Single CPU

The loss or error for the evaluation of U-Net is taken as dice loss, and cross-entropy-based loss is taken as total loss. The cross-entropy has been taken previously for the evaluation of semantic networks. However, the dice loss is primarily taken on recent semantic segmentation network evaluations. By considering both as significant losses, the proposed method takes both as summation by naming the new loss as ‘total loss’. The dice coefficient elaborates the area of prediction compared to the entire area that needs to be predicted. In the proposed method, the loss function calculates using dice and focal loss functions. The calculation of both focal and dice loss is shown in Eqs. (11) and (15).

(11) $$Dice\;Loss = D.L = \;1 - {f_1} - {f_2}$$

(12) $${f_1} = \int_{n = 1}^N \left( {\displaystyle{{({L_{FP}})({L_{FR}}) + \varepsilon } \over {{L_{FP}} + {L_{FR}} + \varepsilon }}} \right)$$

(13) $${f_2} = \int_{n = 1}^N \left( {\displaystyle{{{L_{BP}}*{L_{BR}} + \varepsilon } \over {2 - {L_{FP}} + {L_{FR}} + \varepsilon }}} \right)$$

The dice loss or dice coefficient using *f*_1_ and *f*_2_ is calculated, where *f*_1_ uses a product of the predicted *L*_*FP*_ foreground, and the actual *L*_*FR*_ foreground label masks with the division of both predicted and real foreground masks unite. Additionally, the *ε* weight is added to compensate if the product returns a zero.

(14) $$Cross\;Entropy\;Loss = C.E.\;loss = \;\left\{ {\matrix{ { - log{L_{FP}}\;\;\;\;\;L = 1} \cr { - log{L_{BP}}\;\;\;\;L = 0} \cr } } \right.$$

Cross-entropy loss is a probability measure used for both segmentation and classification, where the more improved form of C.E. loss is Focal Loss (F. Loss), with a modulation factor ${L_{BP}}^\gamma$ of the predicted background label mask value, as shown in Eq. (15).

(15) $$Focal\;Loss = F.\;Loss = \; - {L_{BP}}^\gamma log\;{L_{FP}}$$

The D.E loss and F. loss is used for the segmentation performance measurement, where Total Loss (T. Loss) is shown in Eq. (16).

(16) $$Total\;Loss = T.\;Loss = D.L + {w_t}(F.\;Loss)$$

The predicted and actual masks having both foreground and background pixel counts with an overlapping area division of union for both are calculated as the mean intersection over union (Mean-IoU) for all testing images, as calculated in Eq. (17).

(17) $$Mean\;IoU = \;\int_{I = 1}^N \displaystyle{{Overlapping\;Areas} \over {Union\;of\;Areas}}$$

(18) $$Mean\;F1 - score = \left( {1 + {w_t}} \right)\displaystyle{{\left\{ {Product\left( {Precision,Recall} \right)} \right\}} \over {\{ Union\left( {Precision,Recall} \right)\} }}$$

Another performance measure of precision- and recall-based product and union, with a product of *w*_*t*_ as a constant value, ensures that the value is in a particular range, as calculated in Eq. (18). The total testing loss is 0.17, with a Mean-IoU of 0.79, and the mean F1-score is 0.88. These prediction results are entirely justified, but in some cases, re-consideration is needed. Therefore, quantization is performed to determine exact tumor lesions for more promising breast cancer classification.

### ICA feature-based classification of BUSI data

The extracted features are first given to the extracted 5-fold validation method, which is evaluated in terms of accuracy, precision, recall, f1-score, and kappa statistical index value. The results are shown in Table 4.

**Table 4:** ICA-features-based 5-fold prediction results.

Method	Accuracy (%)	Precision (%)	Recall (%)	F1-Score	Kappa (%)
C-SVM	78.21	81.90	86.96	84.35	48.58
Q-SVM	78.67	80.82	89.70	85.03	48.28
M-Tree	79.91	83.01	88.33	85.59	52.53
R-Boost	80.22	86.87	83.30	85.05	55.86
Bag-Boost	81.92	84.19	90.16	87.07	57.11

**Figure 8:**
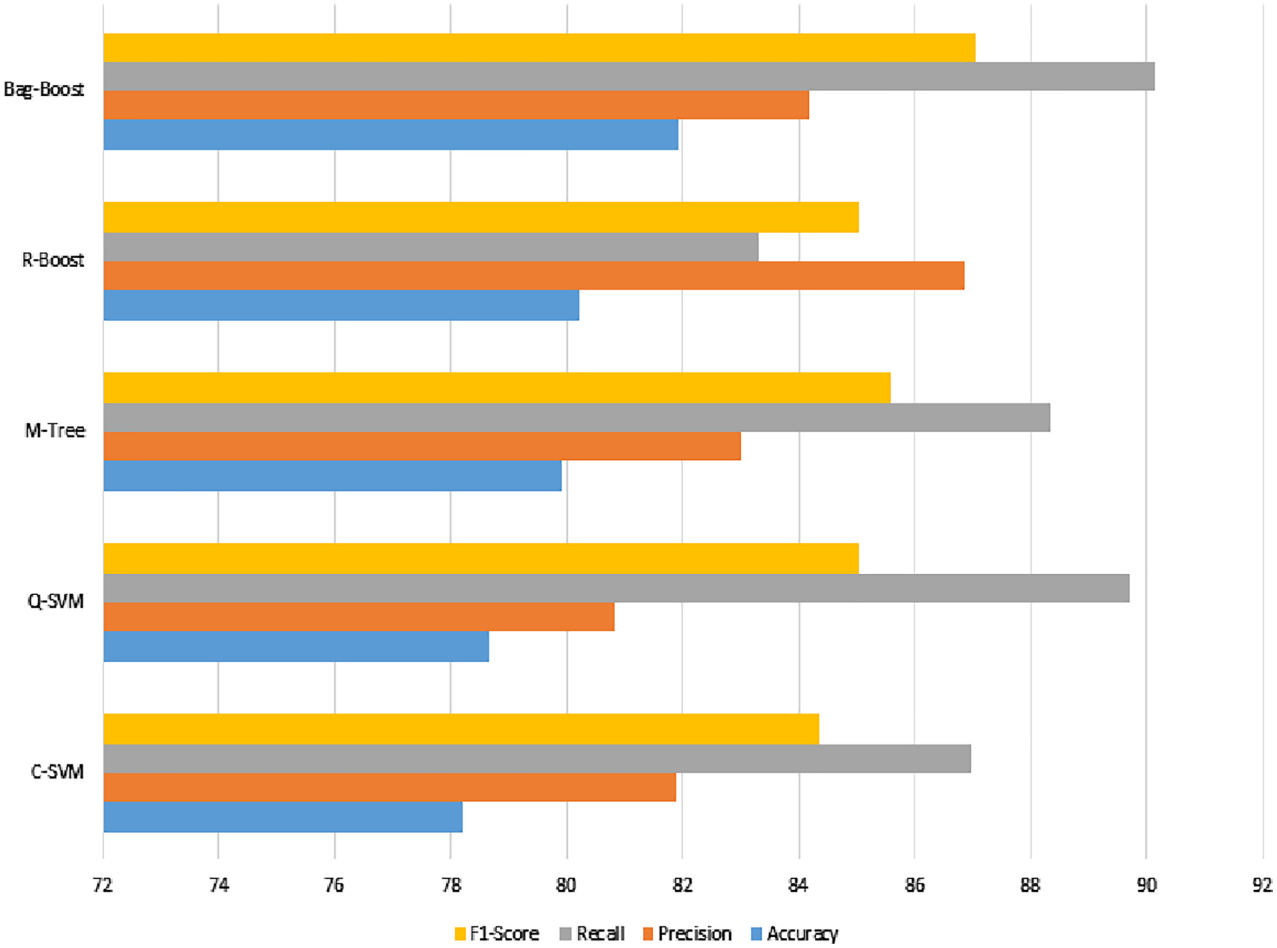
ICA-feature-based classification using a 5-fold validation method, where the accuracy, precision, recall, and F1-score are shown.

The confusion matrix of all methods are combined in one table and shown in Table 5, and a graphical representation of the extracted evaluation measures is shown in Fig. 8. The classification models all reach almost 80%, which is not sufficient. The F1-score, having an effect of both precision and recall, yields approximately 85% for all classifiers. However, the best model using ICA features with a 5-fold validation method is bag-boot in terms of accuracy, and the R-boost is the best among all precision values.

**Table 5:** Confusion matrices of ICA-based 5-fold validation methods.

C-SVM			Q-SVM			M-Tree			R-Boost			Bag-Boost		
Class	B	M	Class	B	M	Class	B	M	Class	B	M	Class	B	M
B	380	57	B	392	45	B	386	51	B	364	73	B	394	43
M	84	126	M	93	117	M	79	131	M	55	155	M	74	136

The best recall value can be considered the most accurate model, bag-boost, which shows a 90.16% value. However, the F1-score based on true positives and false negatives is more considerable for any classification evaluation method. Similarly, the Kappa–Cohen index shows a low level of agreement (McHugh, 2012) for all methods, but bag-boost is the most robust of all of them.

The extracted features are again validated on the mainly used 10-fold cross-validation method, which shows almost the same effect in all measures, with slight changes. The results are shown in Table 6.

**Table 6:** ICA-features-based 10-fold prediction results.

Method	Accuracy (%)	Precision (%)	Recall (%)	F1-Score	Kappa (%)
C-SVM	77.13	80.81	86.73	83.66	45.68
Q-SVM	78.83	81.78	88.33	84.93	49.52
M-Tree	78.36	82.35	86.50	84.38	49.27
R-Boost	79.91	82.45	89.24	85.71	52.04
Bag-Boost	81.61	84.57	89.02	86.73	56.82

**Figure 9:**
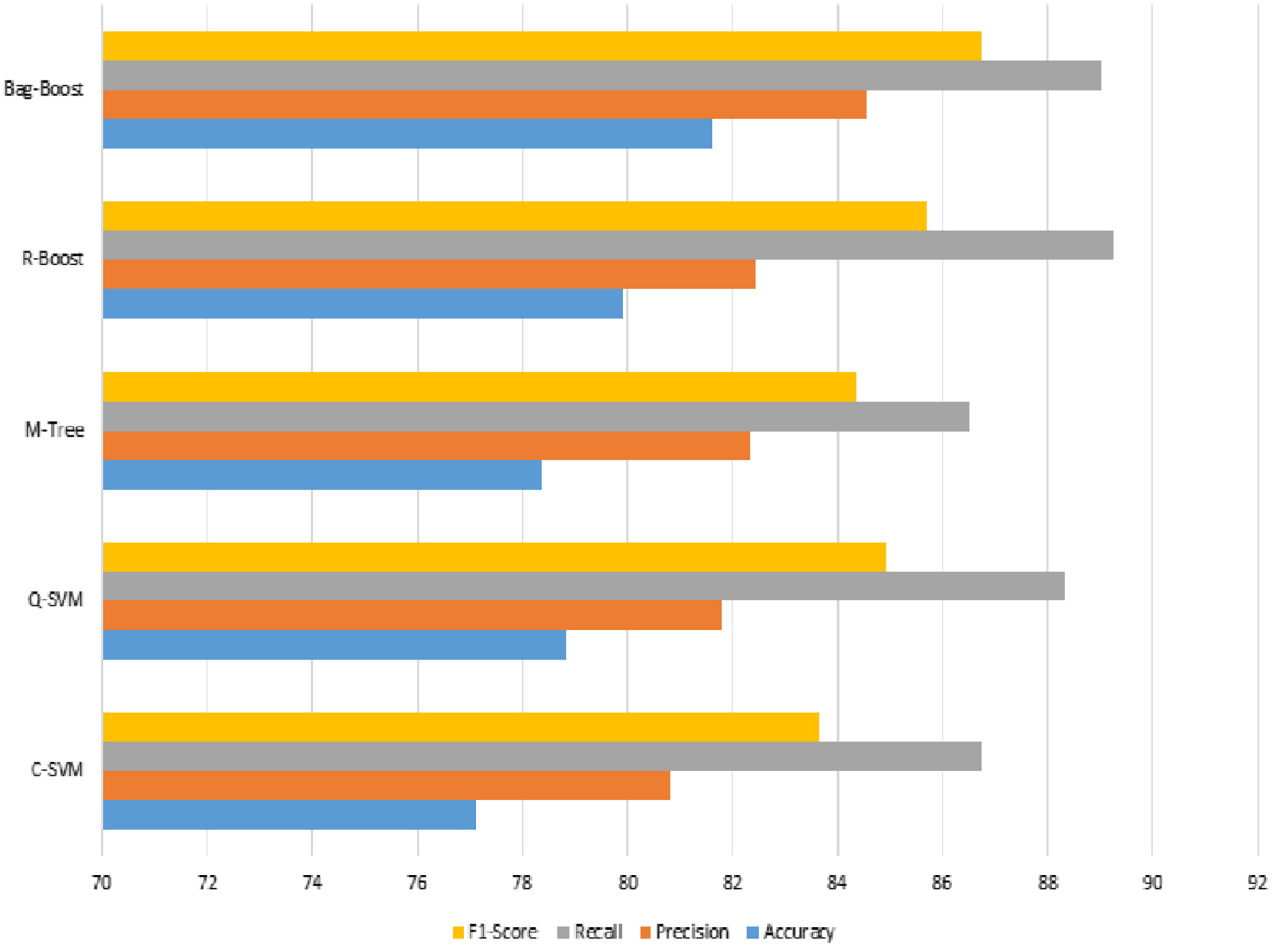
ICA features based classification using 10-fold method where accuracy, precision, recall and F1-score scores have been shown.

The confusion matrix of all methods is combined in one table and shown in Table 6. The visual description is shown in Fig. 9. Tables 5 and 7 comparatively show the effect of the same features and classification methods with fewer and more folds. However, the 5-fold results are better than the 10-fold results, which have fewer chances of bias because they have more folds. Therefore, if we see in Table 7, the bag-boost has fewer wrong predictions than other methods.

**Table 7:** Confusion matrices of ICA-based 10-fold validation methods.

C-SVM			Q-SVM			M-Tree			R-Boost			Bag-Boost		
Class	B	M	Class	B	M	Class	B	M	Class	B	M	Class	B	M
B	379	58	B	386	51	B	378	59	B	390	47	B	389	48
M	90	120	M	86	124	M	81	129	M	83	127	M	71	139

The visualization in Figs. 8 and 9 also show slight changes in cones in all the applied classification methods. However, the ICA-based classification was not so promising, showing less accuracy, so the state-of-the-art method of the deep-learning-based transfer learning of features is applied. These features are then fused with ICA features to check both effects.

### Serial fused results

The deep-transfer-learning-based features can be extracted from many state-of-the-art ImageNet variants that are pre-trained for object classification. The proposed CNN-based transfer learning can also be used to extract deep features. However, denseNet201 has 700+ layers that are dense enough for feature extraction. The extracted deep features are then serially fused with ICA features; the fusion process is already explained by the serial fusion sub-section in the Methodology section. Finally, the fused features are extracted and validated on the aforementioned 5- and 10-fold approaches with the same classification methods.

We can observe from Table 8 that the most accurate method in terms of ICA features changed due to the fusion of both types of features. This is due to the changing range of feature values by the combined effect of both types of features. However, in this case, the best method is Q-SVM, which shows an accuracy of 98.45% with a recall rate of 100%. The F1-score measure with true positives and false negatives is 98.87%, which is also the highest among all classification methods. The confusion matrix of all methods is also improved, as shown in Table 9.

**Table 8:** Serial fusion-based 5-fold prediction results.

Method	Accuracy (%)	Precision (%)	Recall (%)	F1-Score	Kappa (%)
C-SVM	98.15	97.75	99.54	98.64	95.73
Q-SVM	98.45	97.76	100	98.87	96.43
M-Tree	96.45	97.70	97.03	97.36	91.92
R-Boost	97.06	98.16	97.48	97.82	93.33
Bag-Boost	97.99	97.32	99.77	98.53	95.35

**Table 9:** Confusion matrices of fused feature-based 5-fold validation methods.

C-SVM			Q-SVM			M-Tree			R-Boost			Bag-Boost		
Class	B	M	Class	B	M	Class	B	M	Class	B	M	Class	B	M
B	435	2	B	437	0	B	424	13	B	426	11	B	436	1
M	10	200	M	10	200	M	10	200	M	8	202	M	12	198

As per Table 9, the Q-SVM, which gives the highest prediction accuracy, has 0 wrong predictions in the benign class, where the malignant class has 10 wrong predictions. It is clear that the benign class has more training and testing instances, which may lead to the higher amount of accurate prediction results for this class. However, the other class results can also be improved by taking more data.

**Figure 10:**
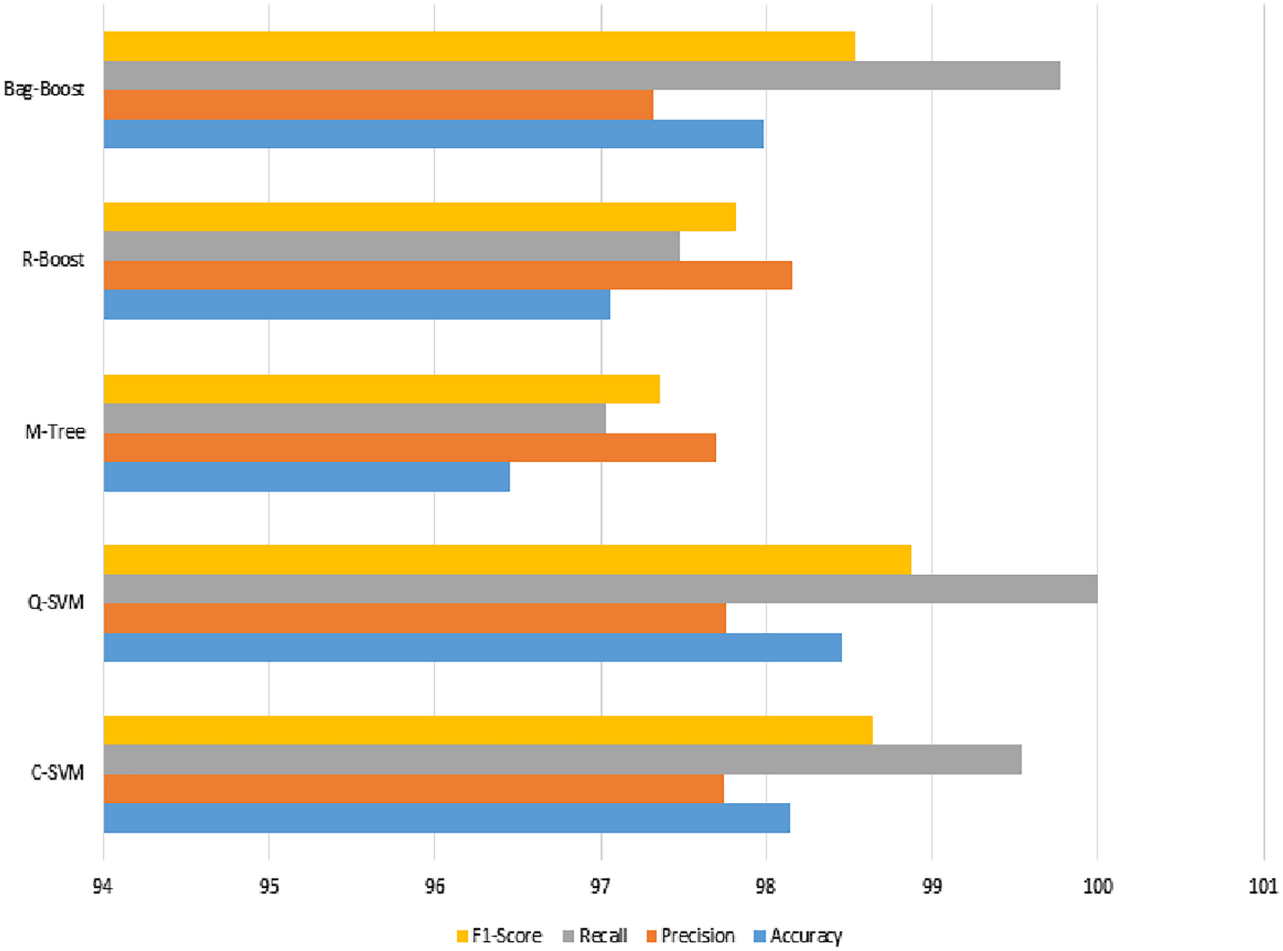
Serial fusion-based 5-fold classification, where the accuracy, precision, recall, and F1-scoreare shown.

The graphical representation in Fig. 10 shows that the C-SVM, Q-SVM, and bag-boost are more accurate than the other classification methods used. These fused features are again validated on 10-fold methods and shown in Table 10.

**Table 10:** Serial fusion-based 10-fold prediction results.

Method	Accuracy (%)	Precision (%)	Recall (%)	F1-Score	Kappa (%)
C-SVM	98.61	98.20	99.77	98.98	96.80
Q-SVM	98.45	97.98	99.77	98.87	96.44
M-Tree	96.10	96.61	97.71	97.16	91.13
R-Boost	97.06	98.16	97.48	97.82	93.33
Bag-Boost	98.15	97.33	100	98.65	95.71

Another validation method is used to confirm the results of the proposed method. The 10-fold classification have more training and testing, and again shows the same good results and an improved performance over other classification methods. If we look into accuracy values comparing 5- and 10-fold classification, the C-SVM is improved values compared to the ICA feature results. The other bag-boost is also improved by 1.14%, where the other precision value is also increased to 100%. Results obtain using serial fusion-based 10-fold prediction are presented in Table 11.

**Table 11:** Serial fusion-based 10-fold prediction results.

C-SVM			Q-SVM			M-Tree			R-Boost			Bag-Boost		
Class	B	M	Class	B	M	Class	B	M	Class	B	M	Class	B	M
B	436	1	B	437	1	B	427	10	B	426	11	B	437	0
M	8	202	M	9	201	M	15	195	M	8	202	M	12	198

The confusion matrix of all methods is reasonable and justified, but the bag-boost has no wrong results in the benign class, and C-SVM and Q-SVM show one wrong prediction. However, the malignant cases are 8 and 9 in C-SVM and Q-SVM, respectively, whereas the bag-boost shows 12 wrong predictions. Therefore, C-SVM is the most accurate in both class predictions compared to the other two models.

**Figure 11:**
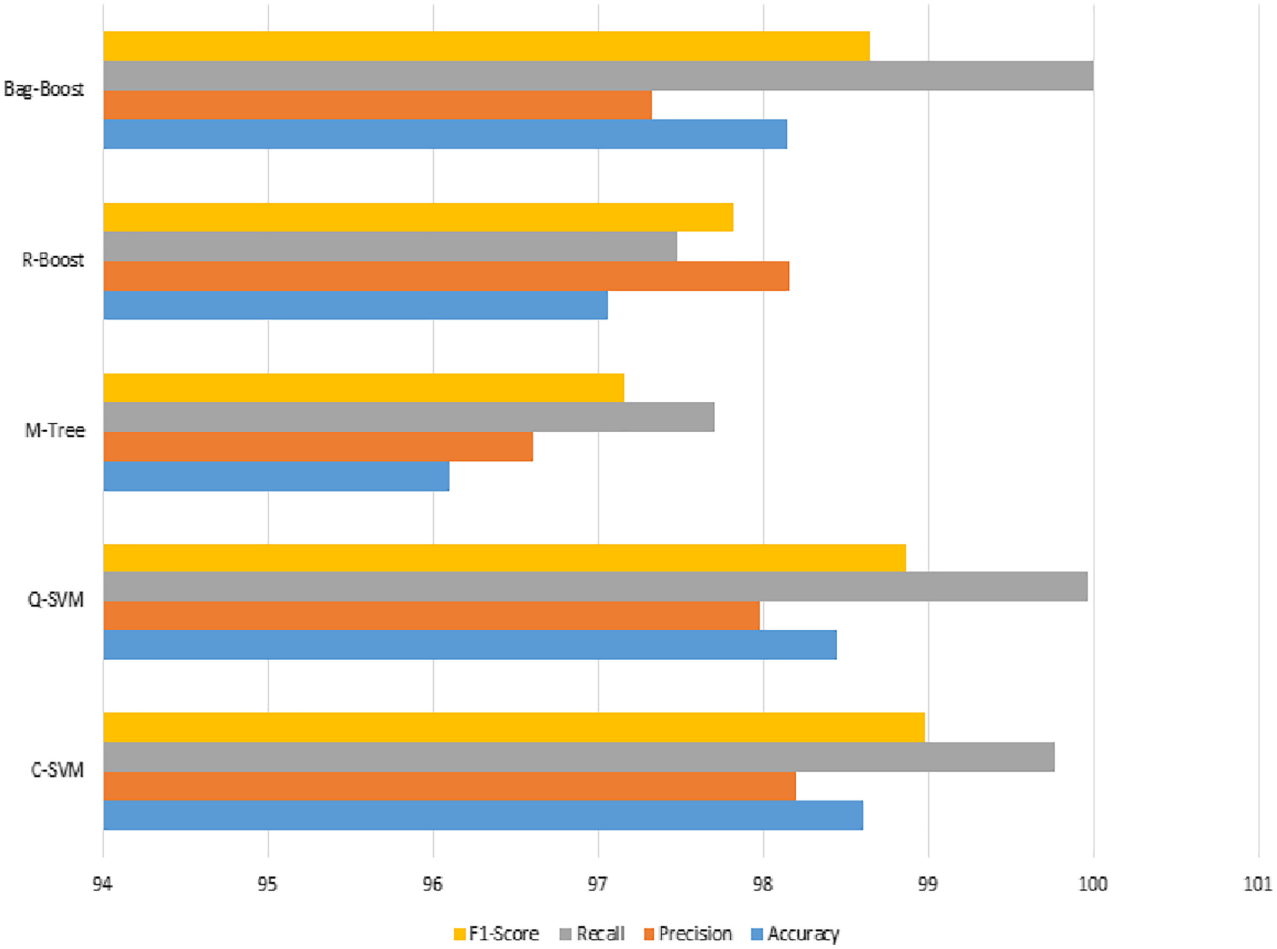
Serial fusion-based classification using a 10-fold method, where the accuracy, precision, recall, and F1-score are shown.

The recall value in Fig. 11 has the highest slope among the best three classification methods of the proposed method, whereas the other M-tree and R-boost have less accurate results in the case of fused features. The other dataset used for testing purposes was also tested on the same features and classification methods. The images were not of the same intensity levels but were of the same modality. Therefore, the same fused features were extracted, but yielded higher results for the feature regularization method of lasso regression. Finally, the regression-based classification was performed and is discussed in the next section.

### Linear regression based OASBUD data classification

The OASBUD segmented data was used by the same feature extraction methods but failed to yield promising results on the classical machine learning methods. Therefore, lasso regularization with a binomial distribution was used on the extracted features for feature selection and classification purposes, where input feature data were fed as a 5- and 10-fold validation method. The results of both validation techniques are justified, as shown in Table 12.

**Table 12:** Lasso regularization-based classification of OASBUD.

Method	Accuracy (%)	Precision (%)	Recall (%)	F1-Score	Kappa (%)
5-fold	94.00	93.75	93.75	93.75	87.98
10-fold	93.00	91.84	93.75	92.78	85.99

The noisy and raw data of the signaling data were used with the same set of features, which also reached a 94% accuracy using feature selection using lasso regularization. For cross-check, the two validation techniques were applied as in previous classical ML methods. The recall, precision, or either kappa index all give strong agreement upon the prediction results.

**Figure 12:**
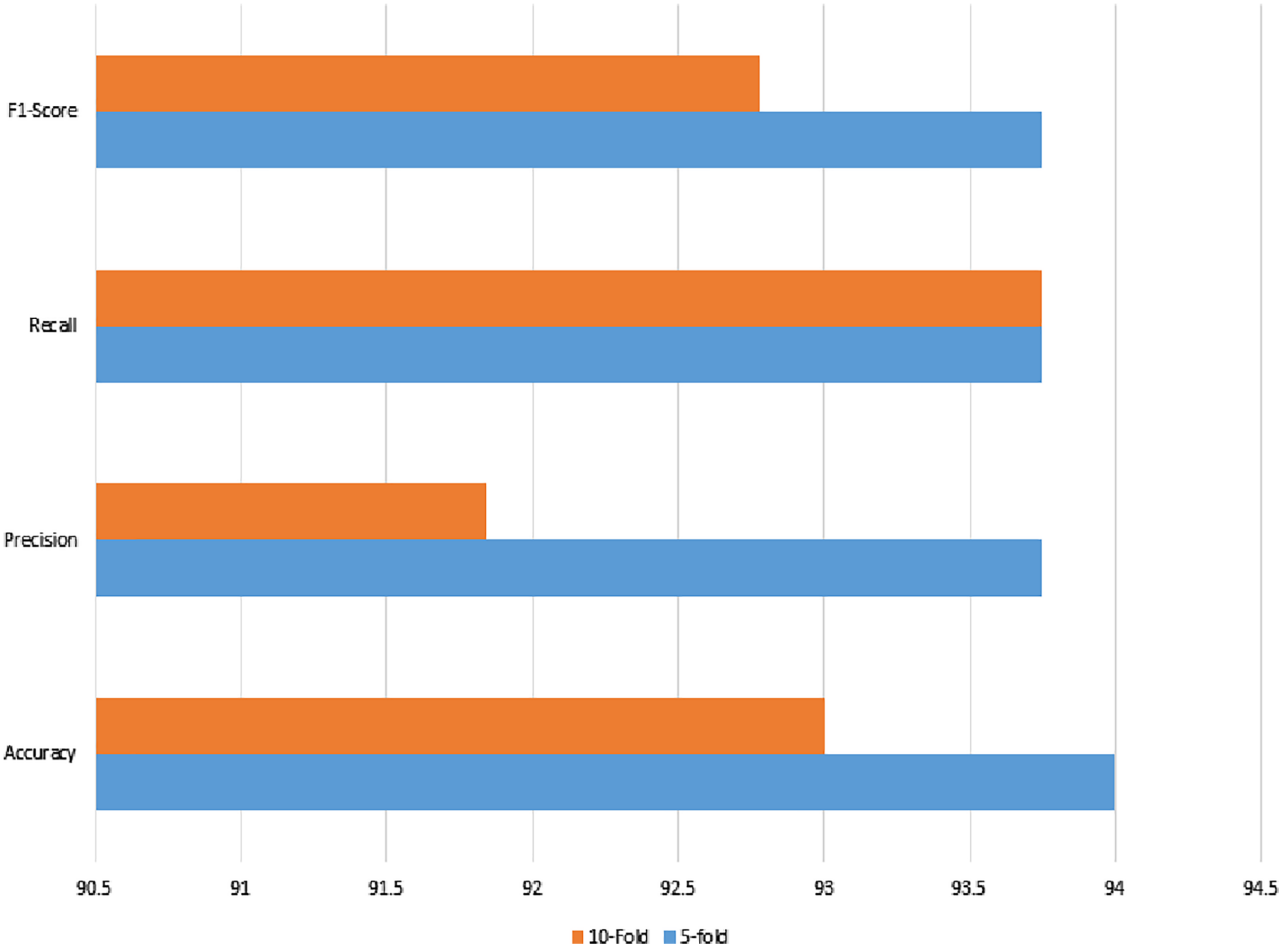
Linear regression method of classification using 5- and 10-fold methods, where the accuracy, precision, recall, and F1-score are shown.

The confusion matrix of both validation methods show that there are three wrong predictions in the case of benign class, whereas there are 3 and 4 wrong predictions in the case of malignant class. The total of 100 instances can also be validated from Table 13. The results are shown in Fig. 12.

**Table 13:** Serial fusion-based 5- and 10-fold prediction results on the OASBUD data.

5-fold			10-fold		
Class	B	M	Class	B	M
B	45	3	B	45	3
M	3	49	M	4	48

The OASBUD dataset was also cross-validated by the ultrasonic dataset with the proposed fused features as a promising method of identifying ultrasonic breast cancer. The proposed method was subsequently compared with state-of-the-art methods to show its superiority.

## Discussion

The proposed method utilizes the semantic deep network approach using the U-Net method, where quantization assists with inaccurately segmented tumors. The ICA features are extracted and tested using 5- and 10-fold validation approaches. The classification results using these features are not promising, as they achieved 81.92% and 81.61% accuracies using 5- and 10-fold classification, respectively. In both methods, the bag-boosting ensemble shows higher accuracy, which means boosting methods of classification are helpful for saliency feature-based recognition. The other type of features using deep, dense features was surprising, as it improves the overall accuracy of all classification methods. Moreover, SVM variant accuracies are high in the case of 5-fold, whereas using 10-fold classification, the bag-boosting method also improves and shows that the bag-boosting method of classification can be used with both types of features; however, the best accuracy using C-SVM is found in the case of the BUSI dataset. If we discuss another testing dataset of OASBUD, the classification methods do not perform well in all classification methods. Therefore, using the same feature extraction methods on the OASBUD dataset, the feature regularization method was adopted. The linear regression model improves its accuracy up to 93% and 94% using 10- and 5-fold validation methods, respectively.

### Comparative analysis

The proposed method was used in different ways to improve accuracy, precision, recall, and F1-score, which was achieved by comparing it with recent studies on the same dataset. The proposed method improved in all ways, including accuracy. These recent studies using various approaches are shown in Table 14.

**Table 14:** Comparison with studies using the same dataset.

References	Method	Accuracy %	Precision (%)	Recall (%)	F1-Score
Moon et al. (2020)	DenseNet-161	94.62	90	92.31	91.14
Moon et al. (2020)	VGG-Like	85.38	75	76.92	75.95
Moon et al. (2020)	DenseNet-121	88.46	77.50	83.78	80.52
Moon et al. (2020)	DenseNet-40	90.77	80	88.89	84.21
Pavithra, Vanithamani & Justin, 2020	Decision tree	85	–	–	–
Pavithra, Vanithamani & Justin, 2020	KNN	83	–	–	–
Pavithra, Vanithamani & Justin, 2020	Random Forest	88	–	–	–
Liu et al. (2020)	SVM	82.69	66.67	93.55	–
Ours	C-SVM	98.61	98.20	99.77	98.98
	Q-SVM	98.45	97.98	99.77	98.87
	M-Tree	96.10	96.61	97.71	97.16
	R-Boost	97.06	98.16	97.48	97.82
	Bag-Boost	98.15	97.33	100	98.65

Table 14 clearly shows that all used classification methods result in improvement as compared to other studies. The compared studies reach a 94.62% accuracy with a 90% precision, a 92.31% recall, and a 91.14% F1-score. The proposed study used five methods that all have an accuracy higher than 96%, and all other measures are also higher than those of the studies compared.

## Conclusion

The proposed research uses ultrasonic-modality-based datasets for breast cancer identification. BUSI is used for U-Net training and testing, whereas OASBUD is employed for testing only. The segmentation results are satisfactory, but some cases can be reconsidered with quantization based on Otsu’s method. The final segmented lesions showed ICA saliency focusing features; however, using deep dense features increases accuracy on the BUSI dataset. The OASBUD dataset shows raw signal data with a low performance on five classical methods. However, feature regularization is used that tends to overcome overfitting problem and enhaced the performance of testing results. Comparative analysis shows the proposed method’s higher performance compared with the current state-of-the-art variants; it can be concluded that the proposed method can be used for ultrasonic breast cancer recognition and identification. The current data used in proposed study have limited data in it where conditional generative adversarial networks can be used in future to generate more data where big data can be the short-coming of proposed study that can be targeted in future. In the future, more datasets can be used, increasing the training for both segmentation and classification. Deep learning for segmentation is also suggested, as it overcame efforts of manually obtaining the valuable features.

## Supplemental Information

10.7717/peerj-cs.805/supp-1 Supplemental Information 1 Code for proposed method.

## Additional Information and Declarations

### Data Availability

The following information was supplied regarding data availability: Dataset 1, "Breast Ultrasound Images Dataset (Dataset BUSI)" is available at https://scholar.cu.edu.eg/?q=afahmy/pages/dataset. Dataset 2 is available at Zenodo: Hanna Piotrzkowska-Wróblewska, Katarzyna Dobruch-Sobczak, Michał Byra, & Andrzej Nowicki. (2017). Open access database of raw ultrasonic signals acquired from malignant and benign breast lesions [Data set]. In Medical Physics. Zenodo. DOI 10.5281/zenodo.545928.
